# Human manipulation strategy when changing object deformability and task properties

**DOI:** 10.1038/s41598-024-65551-x

**Published:** 2024-07-09

**Authors:** A. Mazzeo, M. Uliano, P. Mucci, M. Penzotti, L. Angelini, F. Cini, L. Craighero, M. Controzzi

**Affiliations:** 1 The BioRobotics Institute, Scuola Superiore Sant’Anna, Pisa, Italy; 2 Department of Excellence in Robotics and AI, Scuola Superiore Sant’Anna, Pisa, Italy; 3 Department of Neuroscience and Rehabilitation, University of Ferrara, Ferrara, Italy

**Keywords:** Sensorimotor processing, Neuroscience, Cognitive neuroscience, Biomedical engineering

## Abstract

Robotic literature widely addresses deformable object manipulation, but few studies analyzed human manipulation accounting for different levels of deformability and task properties. We asked participants to grasp and insert rigid and deformable objects into holes with varying tolerances and depths, and we analyzed the grasping behavior, the reaching velocity profile, and completion times. Results indicated that the more deformable the object is, the nearer the grasping point is to the extremity to be inserted. For insertions in the long hole, the selection of the grasping point is a trade-off between task accuracy and the number of re-grasps required to complete the insertion. The compliance of the deformable object facilitates the alignment between the object and the hole. The reaching velocity profile when increasing deformability recalls the one observed when task accuracy and precision decrease. Identifying human strategy allows the implementation of human-inspired high-level reasoning algorithms for robotic manipulation.

## Introduction

Humans routinely interact with rigid and non-rigid objects^1,2^. On the contrary, general methods to effectively model, control, and perceive the state of non-rigid objects for robotic manipulation are currently debated topics in research^2,3^. Industrial assembly tasks, or also domestic or assistive activities that require the use of deformable objects are nowadays mainly performed by humans^4,5^.

Human manipulation behavior has been widely studied to date. Features like kinematics of the reach-to-grasp movement^6^, grasp type, location, and dimensions^7–9^ have been characterized, with the aim of either simply describing human behavior or transferring human strategy to the robotic hardware. Datasets describing object manipulation, eventually including also everyday-life deformable objects, were published^10^ and became standards for robotic grasping and manipulation algorithm training or benchmarking^11^. However, no study has focused on analyzing the influence that the different levels of deformability of the object have on human movement kinematics and manipulation strategy.

Only a few studies on humans manipulating exclusively deformable objects have been performed, focusing on specific applications, such as tomato fruit-picking^12^, and folding clothes^13^. Other studies concerning human manipulation of generic objects^14^ found some differences between the grasps executed for non-rigid objects and the grasps identified in the taxonomy of human grasp types^15^. The grasp types mentioned specifically for non-rigid objects in housekeeping tasks were power sphere and precision disk^14,15^. In the analysis of human grasp behavior by Feix et al.^7^, fragile, squeezable, and floppy objects were distinguished from rigid ones, and interestingly, when discussing properties like object shape, dimension, or roundness, floppy objects were not accounted for.

Other approaches to studying human manipulation of deformable objects consist in modeling them as non-rigid objects, i.e., cart-pendulum systems to model coffee displacement in a cup^16^ or floppy objects as mass-spring systems^17,18^. In these studies, authors tackled the problem of how humans control the position of specific points of the object that are not rigidly attached to the hand. In natural object manipulation tasks, we can exert control over numerous locations, namely “control points” on the object^19^. When drinking from a wine glass, for example, we can control the near rim (which we can define as control point n. 1) as we lift the glass to our mouth, and then the base of the stem (control point n. 2) as we replace the glass. This control applies to deformable objects as well, for example when we spread a towel over the sand, controlling the position of the furthest corners by handling the nearest ones. The authors found that smoothness of human motion is not related only to hand point-to-point motion^17^, hypothesizing that planning accounts for an internal model^1,20,21^ of the forces to be exerted by the hand on the object. The results of a psychophysical study in humans^19^, using a planar robotic interface and virtual-reality system to apply opposing viscous curl fields to two control points on a virtual object, suggested that learning of the dynamics of objects (i.e. the motor memory of the interaction) is linked to control points on the object, rather than to the object itself. The same reasoning, in principle, might apply to each control point on a deformable object that might be associated with different (and not constant) dynamics.

The aim of the present study is to investigate the influence of different levels of object deformability on the grasping behavior adopted by humans to perform a given task^9,22^ when different degrees of accuracy and different end-goals are required. End-goals were also named subsequent tasks^9,23^, and they were proven to impact the reaching and grasping behavior. In addition to giving novel insights to the kinematics of human movement during interaction with deformable objects, the results of the experiment will allow the development of human-inspired high-level reasoning algorithms for robotic manipulation.

**Figure 1:**
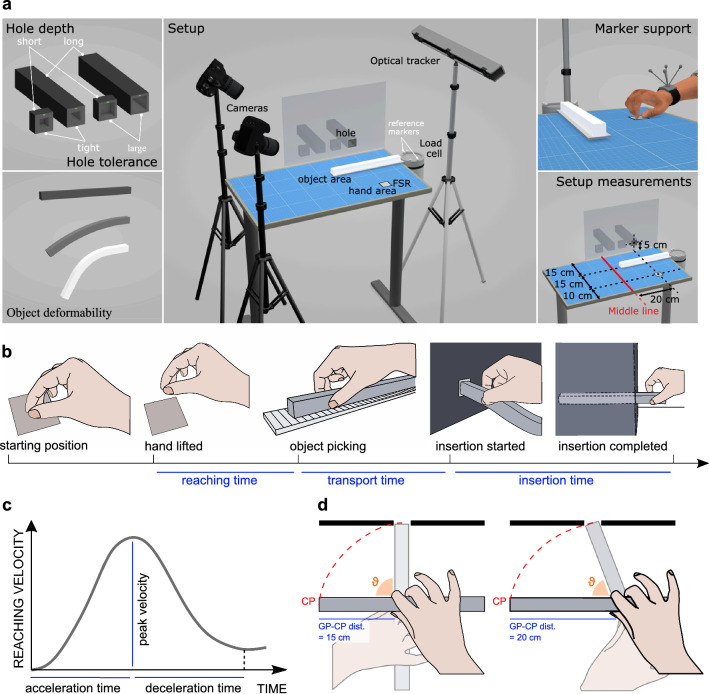
Schema of the experimental setup and measured variables. (**a**) Schema of the experimental setup, including representations of the short and long, tight and large holes, the deformability of the objects, and the marker support for motion tracking. (**b**) Graphical representation of the measured time intervals. (**c**) Reaching velocity parameters. (**d**) Representation of the trajectory of the control point (CP) for different grasping point—control point (GP–CP) distances: the CP follows the red dashed path during the transport phase; hence, when the GP–CP distance is shorter, a higher hand rotation is required to span the same path; in the long large hole condition the GP–CP distance was higher for the rigid and intermediate objects, so hypothesizing participants moved as fast as possible as required, the smaller rotation performed might explain the shorter transport time for those two objects.

In the present study, participants were required to fully insert three long parallelepipeds with different levels of deformability (rigid, intermediate, deformable) into holes of different sizes (tight, large) and different lengths (short, long), as illustrated in Fig. 1a. Participants were asked to complete this task as fast as possible. We have divided the duration of the entire movement into phases (Fig. 1b), considering the duration of the movement to reach the object (reaching time), of the transport of the object up to the inlet of the hole (transport time), of the insertion of the object into the hole (insertion time). We considered the distance between the point of contact of the fingers with the object (grasping point, GP), the end of the object to be introduced into the hole (control point, CP), and the number of re-grasps needed to fully insert the object (after the first grasp). Finally, we performed the kinematic analysis of the reaching movement (Fig. 1c): from the reaching velocity profile, we retrieved the peak velocity, and acceleration and deceleration times.

Results suggested that the selected GP is a trade-off between the deformability of the object and the insertion depth. In addition, only for long-hole insertion, the GP and the number of re-grasps vary also with accuracy. Interestingly, the participants who were the fastest in manipulating the object were also those who adopted the most commonly used GP and number of re-grasps. The alignment of the CP with the hole resulted to be easier for the deformable object since the remaining part of the object passively followed the movement. For the rigid object instead, the participant needs to control the movement of the object as a whole to avoid potential collisions with the environment. Moreover, when increasing the deformability, the kinematics of the reaching phase resembled the results obtained when reaching to execute a task with lower accuracy requirements.

## Results

### Reaching, transport, and insertion time

**Figure 2:**
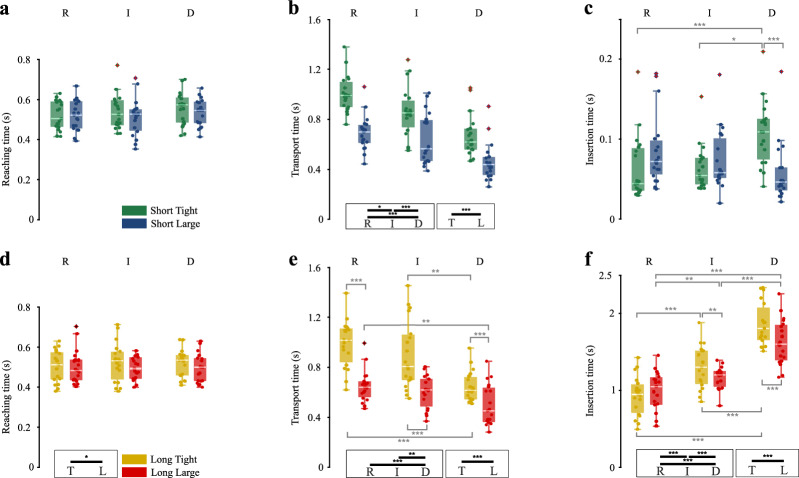
Reaching, transport, and insertion time for insertions in the short and long holes. Boxplots show the distribution (median, IQR, max/min value, outliers) of the mean time for the short- and long-hole insertion for each participant across the conditions of hole tolerance (T tight, L large) and object deformability (R rigid, I intermediate, D deformable). The dots represent the mean reaching, transport, and insertion time for short- and long-hole insertion for each participant in a specific condition. Horizontal bars represent significant differences obtained with post-hoc tests, in black between deformability or accuracy levels (within the boxes), in gray between experimental conditions; asterisks refer to the Bonferroni adjusted *p*-values within the following ranges: * for *p*_*adj*_ < 0.05, ** for *p*_*adj*_ < 0.01, *** for *p*_*adj*_ < 0.001. (**a**) Mean reaching time, (**b**) Mean transport time, and (**c**) Mean insertion time for the short-hole insertion. (**d**) Mean reaching time, (**e**) Mean transport time, and (**f**) Mean insertion time for the long-hole insertion.

Figure 2 shows the time duration of the reaching, transport, and insertion phases for the short- and long-hole insertions, and the results of the statistical analysis, whose details are reported in Table 1. In this paragraph, *p*-values refer to two-way repeated measures ANOVA, unless otherwise noted, and *p*_*adj*_ refers to *p*-values computed with Bonferroni adjustment for multiple comparisons. Reaching time is the duration from the moment the hand is lifted to object picking, transport time is the duration from object picking to the beginning of the insertion, and insertion time is the duration from the beginning to the end of the insertion (Fig. 1b).

**Table 1:** Statistical results for reaching, transport, and insertion time, and grasping point—control point (GP–CP) distance. Results of ANOVA tests (F) and its post-hoc comparisons, and results of the Friedman tests (χ^2^) and its post-hoc comparisons with T-Wilcoxon Signed Ranks test (^W^) or Sign test (^S^). For the post-hoc test, *p*-values (*p*_*adj*_) are adjusted with the Bonferroni correction. Significant results (*p* < 0.05) are highlighted in boldface. *R* Rigid object, *I* Intermediate object, *D* Deformable object, *L* Large hole, *T* Tight hole.

				Reaching		Transport		Insertion		GP–CP distance	
				Short hole	Long hole	Short hole	Long hole	Short hole	Long hole	Short hole	Long hole
				N = 19	N = 19	N = 19	N = 19	N = 19	N = 19	N = 19	N = 19
Main effects	Deformability			F = 2.745 *p* = 0.078	F = 0.221 *p* = 0.803	**F = 35.186** ***p* < 0.001**	**F = 24.587** ***p* < 0.001**	χ^2^ = 2.632 *p* = 0.268	**F = 95.421** ***p* < 0.001**	**χ**^**2**^** = 34.105** ***p* < 0.001**	**χ**^**2**^** = 38.000** ***p* < 0.001**
	Accuracy			F = 1.856 *p* = 0.190	**F = 4.461** ***p* = 0.049**	**F = 101.834** ***p* < 0.001**	**F = 119.513** ***p* < 0.001**	Sign test *p* = 0.359	**F = 20.842** ***p* < 0.001**	Sign test *p* = 0.359	**Sign test** ***p* < 0.001**
	Deformability*Accuracy			F = 0.830 *p* = 0.444	F = 1.438 *p* = 0.251	F = 2.333 *p* = 0.112	**F = 4.804** ***p* = 0.025**	**χ**^**2**^** = 22.842** ***p* < 0.001**	**F = 13.329** ***p* < 0.001**	χ^2^ = 0.105 *p* = 0.949	**χ**^**2**^** = 18.105** ***p* < 0.001**
Post-hoc	Deformability	R–I		–	–	***p***_***adj***_** = 0.032**	*p*_*adj*_ = 0.215	–	***p***_***adj***_** < 0.001**	***p***_***adj***_** < 0.001**^W^	***p***_***adj***_** < 0.001**^**W**^
		R–D		–	–	***p***_***adj***_** < 0.001**	***p***_***adj***_** < 0.001**	–	***p***_***adj***_** < 0.001**	***p***_***adj***_** < 0.001**^S^	***p***_***adj***_** < 0.001**^**W**^
		I–D		–	–	***p***_***adj***_** < 0.001**	***p***_***adj***_** = 0.001**	–	***p***_***adj***_** < 0.001**	***p***_***adj***_** < 0.001**^S^	***p***_***adj***_** < 0.001**^**W**^
	Deformability*Accuracy	Tight	R–I	–	–	–	*p*_*adj*_ = 0.524	*p*_*adj*_ = 1.000^S^	***p***_***adj***_** < 0.001**	–	***p***_***adj***_** < 0.001**^**S**^
			R–D	–	–	–	***p***_***adj***_** < 0.001**	***p***_***adj***_** = 0.013**^S^	***p***_***adj***_** < 0.001**	–	***p***_***adj***_** < 0.001**^**S**^
			I–D	–	–	–	***p***_***adj***_** = 0.002**	***p***_***adj***_** < 0.001**^**S**^	***p***_***adj***_** < 0.001**	–	***p***_***adj***_** = 0.016**^**W**^
		Large	R–I	–	–	–	*p*_*adj*_ = 0.569	*p*_*adj*_ = 1.000^S^	***p***_***adj***_** = 0.005**	–	***p***_***adj***_** < 0.001**^**W**^
			R–D	–	–	–	***p***_***adj***_** = 0.007**	*p*_*adj*_ = 0.057^S^	***p***_***adj***_** < 0.001**	–	***p***_***adj***_** < 0.001**^**W**^
			I–D	–	–	–	*p*_*adj*_ = 0.095	*p*_*adj*_ = 0.057^S^	***p***_***adj***_** < 0.001**	–	***p***_***adj***_** < 0.001**^**W**^
		R	L–T	–	–	–	***p***_***adj***_** < 0.001**	*p*_*adj*_ = 0.064^S^	*p*_*adj*_ = 0.084	–	***p***_***adj***_** < 0.001**^**S**^
		I	L–T	–	–	–	***p***_***adj***_** < 0.001**	*p*_*adj*_ = 0.064^S^	***p***_***adj***_** = 0.006**	–	***p***_***adj***_** < 0.001**^**W**^
		D	L–T	–	–	–	***p***_***adj***_** < 0.001**	***p***_***adj***_** < 0.001**^S^	***p***_***adj***_** < 0.001**	–	***p***_***adj***_** = 0.001**^**W**^

#### Reaching time

As for the reaching time, results showed no significant effect of either accuracy or deformability in the short-hole insertion (Fig. 2a). A slightly significant effect of the accuracy (*p* = 0.049) was reported instead for the long-hole insertion (Fig. 2d).

#### Transport time

As for the transport time, results showed a statistically significant effect of the deformability for both the short- and long-hole insertion (*p* < 0.001, Fig. 2b,e respectively). The transport time decreased with the increasing deformability, showing significant differences among the time needed to transport the deformable object and the time needed to transport the intermediate and rigid ones (*p*_*adj*_ ≤ 0.001). For the short hole (Fig. 2b), the time needed to transport the rigid object and the intermediate one was significantly different as well (*p*_*adj*_ = 0.032). A significant effect of the accuracy (*p* < 0.001) was reported for both the short- and long-hole insertion. For the long-hole insertion (Fig. 2e), the interaction between deformability and accuracy was also significant (*p* = 0.025): for each level of deformability, the transport time in the tight hole was longer than the respective in the large hole (*p*_*adj*_ < 0.001). For both accuracies, the time for transporting the rigid object was significantly longer than the time to transport the deformable one (tight *p*_*adj*_ < 0.001, large *p*_*adj*_ = 0.007); for the tight hole, the time to transport the intermediate object was also significantly longer than the time to transport the deformable one (*p*_*adj*_ = 0.002).

#### Insertion time

As for the insertion time, deformability and accuracy factors had no significant effect in the short-hole insertion (Fig. 2c). Their interaction was significant instead (Friedman test, *p* < 0.001): for the tight-hole insertion, the time to insert the deformable object was significantly longer than the time needed to insert the rigid and intermediate ones (Sign tests, *p*_*adj*_ = 0.013, and *p*_*adj*_ < 0.001); the insertion time for the deformable object for the large-hole insertion was instead significantly shorter than the one for the tight-hole insertion (Sign test, *p*_*adj*_ < 0.001). On the other hand, in the long-hole insertion (Fig. 2f), deformability, accuracy, and their interaction all had a significant effect on the insertion time (*p* < 0.001): post-hoc tests confirmed that insertion time differed among each level of deformability. For intermediate and deformable objects, post-hoc tests also showed significant differences between insertion time in the large-hole and tight-hole insertions (intermediate *p*_*adj*_ = 0.006, deformable *p*_*adj*_ < 0.001).

### Distance between grasping point and control point, and number of re-grasps

**Figure 3:**
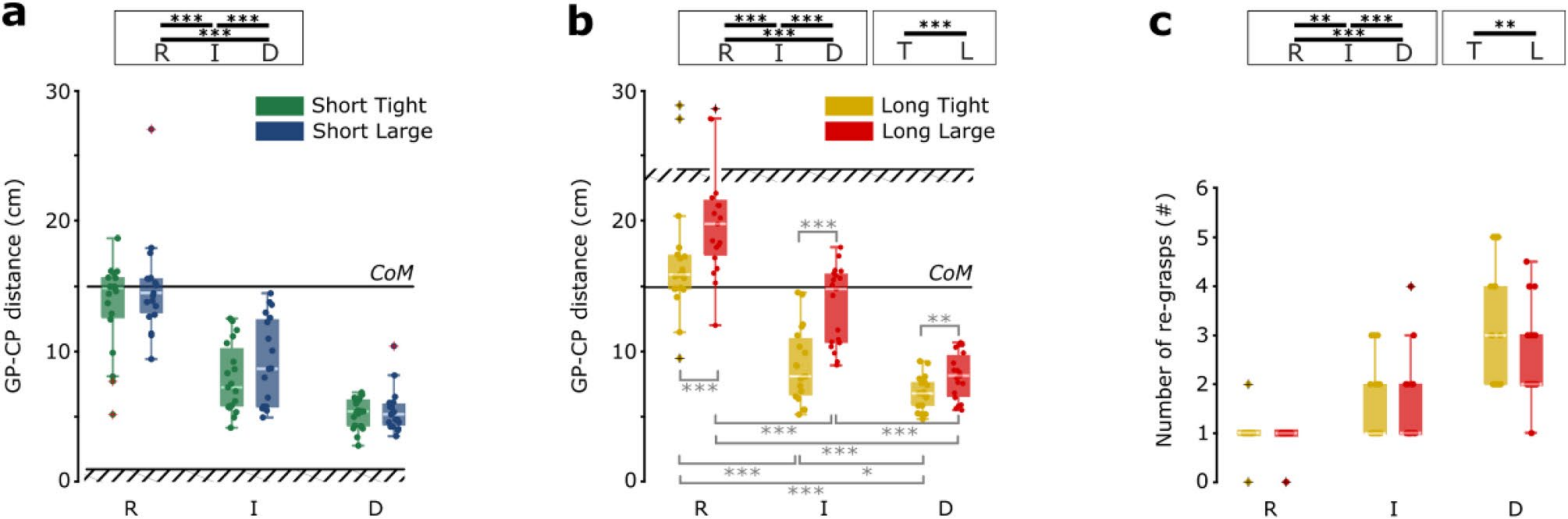
Distance between grasping point (GP) and control point (CP), and the number of re-grasps. (**a**,**b**) Boxplots show the distribution (median, IQR, max/min value, outliers) of the mean GP–CP distance for the short-hole and long-hole insertion for each participant across the conditions of hole tolerance (*T* tight, *L* large) and object deformability (*R* rigid, *I* intermediate, *D* deformable). The dots represent the mean GP–CP distance for short- and long-hole insertion for each participant in a specific condition. (**c**) Boxplots show the distribution (median, IQR, max/min value, outliers) of the median number of re-grasps for the long-hole insertion for each participant across the conditions of hole tolerance (*T* tight, *L* large) and object deformability (*R* rigid, *I* intermediate, *D* deformable). The dots represent the median number of re-grasps for the long-hole insertion for each participant in a specific condition. The lines labeled with *CoM* denote the center of mass of the object, while the patterned lines remark the depth of the holes. Horizontal bars represent significant differences obtained with post-hoc tests, in black between deformability or accuracy levels (within the boxes), in gray between experimental conditions; asterisks refer to the Bonferroni adjusted *p*-values within the following ranges: * for *p*_*adj*_ < 0.05, ** for *p*_*adj*_ < 0.01, *** for *p*_*adj*_ < 0.001.

Figure 3 shows the GP–CP distance, that is the distance between the GP (the point of contact of the fingers with the object) and the CP (the extremity of the object to be introduced into the hole), for the short- and long-hole insertion, and the number of re-grasps for the long-hole insertion. Figure 3 also shows the results of the statistical analysis (non-parametric tests), and more details are reported in Table 1 for the distance and Table 2 for the number of re-grasps.

**Table 2:** Statistical results for number of re-grasps, wrist peak velocity, acceleration and deceleration time rates. Results of ANOVA tests (F) and its post-hoc comparisons, and results of the Friedman tests (χ^2^) and its post-hoc comparisons with T-Wilcoxon Signed Ranks test (^W^) or Sign test (^S^). For the post-hoc test, *p*-values (*p*_*adj*_) are adjusted with the Bonferroni correction. Significant results (*p* < 0.05) are highlighted in boldface. *R* Rigid object, *I* Intermediate object, *D* Deformable object.

			# Re-grasps	Peak velocity		Acceleration time rate		Deceleration time rate	
			Long hole	Short hole	Long hole	Short hole	Long hole	Short hole	Long hole
			N = 19	N = 17	N = 17	N = 17	N = 17	N = 17	N = 17
Main effects	Deformability		**χ**^**2**^** = 35.565** ***p* < 0.001**	**F = 7.478** ***p* = 0.002**	**F = 7.536** ***p* = 0.006**	**F = 5.170** ***p* = 0.011**	**F = 5.279** ***p* = 0.010**	**F = 5.170** ***p* = 0.011**	**F = 5.279** ***p* = 0.010**
	Accuracy		**Sign test** ***p* = 0.004**	F = 0.246 *p* = 0.627	F = 2.433 *p* = 0.138	F = 2.630 *p* = 0.124	F = 2.301 *p* = 0.149	F = 2.630 *p* = 0.124	F = 2.301 *p* = 0.149
	Deformability *Accuracy		χ^2^ = 3.050 *p* = 0.218	F = 1.109 *p* = 0.342	F = 1.545 *p* = 0.229	F = 1.101 *p* = 0.345	F = 0.916 *p* = 0.410	F = 1.101 *p* = 0.345	F = 0.916 *p* = 0.410
Post-hoc tests	Deformability	R–I	***p***_***adj***_** = 0.001**^S^	*p*_*adj*_ = 1.000	*p*_*adj*_ = 0.185	*p*_*adj*_ = 0.131	*p*_*adj*_ = 1.000	*p*_*adj*_ = 0.131	*p*_*adj*_ = 1.000
		R–D	***p***_***adj***_** < 0.001**^S^	***p***_***adj***_** = 0.009**	***p***_***adj***_** = 0.022**	***p***_***adj***_** = 0.032**	***p***_***adj***_** = 0.004**	***p***_***adj***_** = 0.032**	***p***_***adj***_** = 0.004**
		I–D	***p***_***adj***_** < 0.001**^S^	*p*_*adj*_ = 0.052	*p*_*adj*_ = 0.071	*p*_*adj*_ = 1.000	*p*_*adj*_ = 0.080	*p*_*adj*_ = 1.000	*p*_*adj*_ = 0.080

#### Distance between grasping point and control point

Deformability had a significant effect on the GP–CP distance (Friedman test, *p* < 0.001), and post-hoc tests showed significant differences among all deformability levels for the insertions in both short and long holes (*p*_*adj*_ < 0.001 at least, see details in Table 1): the GP–CP distance decreased with the deformability. Accuracy had no significant effect on the GP–CP distance for the short-hole insertion (Fig. 3a), while it was significant for the long-hole insertion (T-Wilcoxon Signed Ranks test, *p* < 0.001, Fig. 3b). The interaction between deformability and accuracy was significant only for the long-hole insertion, and all post-hoc comparisons among the conditions resulted in significant differences (*p*_*adj*_ ≤ 0.016 at least, see details in Table 1): the GP was near the center of mass when the object is rigid, and it shifted toward the CP with object deformability. GP shifted instead far from the CP for the low insertion accuracy.

Overall, considering all the insertions performed by all the participants, the maximum GP–CP distance used for the intermediate object (without considering outliers) was: 205 mm for the long large hole; 160 mm for the long tight hole; 170 mm for the short large hole; and 155 mm for the short tight hole. For the deformable object, the maximum GP–CP distance used (without considering outliers) was: 135 mm for the long large hole; 115 mm for the long tight hole; 115 mm for the short large hole; and 90 mm for the short tight hole.

#### Number of re-grasps

Regarding the number of re-grasps, for the short hole, in any condition every participant used a single grasp (except for one single trial), meaning re-grasps were never performed for the short hole. For the long-hole insertion (Fig. 3c), deformability had a significant effect on the number of re-grasps, which increased with increasing deformation (Friedman test, *p* < 0.001), and post-hoc tests showed significant differences among all deformability levels (Sign test, *p*_*adj*_ ≤ 0.001 at least, see details in Table 2).

#### Most frequently chosen strategies

Figure S1 (Supplementary materials), illustrates the strategies most frequently chosen by the participants for the long-hole insertion, and Table SI (Supplementary materials), the strategy adopted by the fastest participants. Among the combinations of number of re-grasps and GP–CP distance, the most frequent choice for the rigid object was to use 1 re-grasp, for the tight hole (15/19 participants) with a mean GP near the center of mass (155 mm ± 25 mm), and for the large hole (16/19 participants) with a slightly farther GP (185 mm ± 25 mm). For the intermediate object, the most frequent choice was to use 1 re-grasp for the tight hole (11/19 participants) with a mean GP of about 90 mm ± 30 mm, and 1 re-grasp for the large hole (12/19 participants) nearer to the center of mass (140 mm ± 30 mm). For the deformable object, the most frequent choice was to use 2 re-grasps for the tight hole (9/19 participants) with a mean GP of around 70 mm ± 15 mm, and 2 re-grasps for the large hole (10/19 participants) with a mean GP of around 80 mm ± 15 mm. Comparing the ranges of GP–CP distances of the intermediate and deformable objects for the participants who chose the most frequent strategy (Fig. S1b), the dispersion resulted higher for the intermediate one (Kruskal–Wallis dispersion test, χ^2^ = 6.870 *p* = 0.009 for the tight hole, χ^2^ = 4.452 *p* = 0.035 for the large hole).

### Kinematic data of the reaching movement

**Figure 4:**
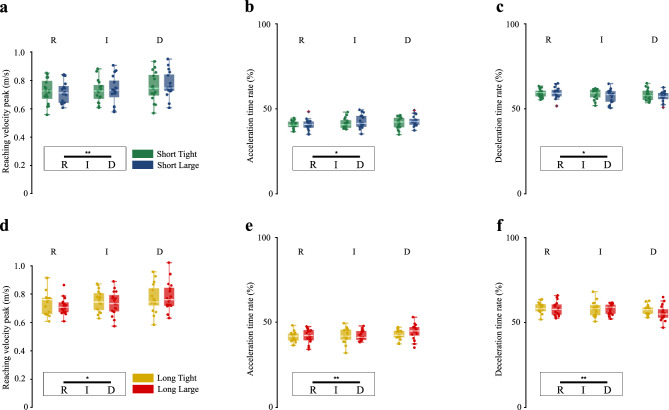
Parameters from wrist reaching velocity profile: peak velocity (m/s), and acceleration and deceleration time rates (%), for insertions in the short and long holes. Boxplots show the distribution (median, IQR, max/min value, outliers) of the mean reaching kinematic parameters for the short- and long-hole insertion for each participant across the conditions of hole tolerance (*T* tight, *L* large) and object deformability (*R* rigid, *I* intermediate, *D* deformable). The dots represent the mean peak velocity, acceleration and deceleration time rates, for the short- and long-hole insertion for each participant in a specific condition. Horizontal bars represent significant differences obtained with post-hoc tests, in black between deformability or accuracy levels (within the boxes), in gray between experimental conditions; asterisks refer to the Bonferroni adjusted *p*-values within the following ranges: * for *p*_*adj*_ < 0.05, ** for *p*_*adj*_ < 0.01, *** for *p*_*adj*_ < 0.001. (**a**) Mean peak velocity, (**b**) Mean acceleration time rate, and (**c**) Mean deceleration time rate for the short-hole insertion. (**d**) Mean peak velocity, (**e**) Mean acceleration time rate, and (**f**) Mean deceleration time rate for the long-hole insertion.

Figure 4 shows the peak wrist velocity during the reaching phase, and the acceleration and deceleration time rates for the short- and long-hole insertion; it also shows the results of the statistical analysis (in this paragraph, *p*-values refer to two-way repeated measures ANOVA, and *p*_*adj*_ refers to *p*-values computed with Bonferroni adjustment for multiple comparisons), whose details are reported in Table 2. Deformability had a significant effect on peak velocity (Fig. 4a,d), and on acceleration (Fig. 4b,e) and deceleration (Fig. 4c,f) time rates (at least *p* ≤ 0.011, see details in Table 2). Post-hoc comparisons showed a significant difference between rigid and deformable objects for peak velocity (higher for the deformable object), acceleration (greater for the deformable object) and deceleration (smaller for the deformable object) time rates (at least *p*_*adj*_ ≤ 0.032, see details in Table 2).

Figure S2 (Supplementary materials) shows the reaching time, acceleration and deceleration times extracted from the wrist velocity profile for the short- and long-hole insertion; it also shows the results of the statistical analysis (two-way repeated measures ANOVA), whose details are reported in Table SII, Supplementary materials. In both short- (Fig. S2a) and long-hole (Fig. S2d) conditions, the duration of the reaching phase did not show variations with deformability (the deformability factor is significant at *p* = 0.041, but there is no significance in post-hoc comparisons, see details in Table SII, Supplementary materials) or accuracy. There were no significant differences in acceleration time (Fig. S2b,e). Only in the short-hole condition, there was an effect of deformability on deceleration time (Fig. S2c), and post-hoc comparisons showed a significant difference between rigid and deformable objects (*p*_*adj*_ = 0.036, see details in Table SII, Supplementary materials).

## Discussion

### The grasping point reflects the compromise between task accuracy and the number of re-grasps

The GP for rigid objects is selected near the center of mass (CoM) of the objects, to avoid additional torques leading to object rotations^24^, and CoM is estimated both visually and haptically^25^. Moreover, the GP is influenced by other object properties such as size^26^, shape, weight, and friction coefficient^27^, and other factors such as object location and orientation with respect to the natural grasping axis^28^, the direction of the movement, and participant’s experience, maximization of object visibility^27,28^ and task to be performed^9^.

Present results show that the GP for the rigid object was near the CoM according to the literature^24,25^ for the short-hole insertion, regardless of the level of accuracy required (Fig. 3a). The GP shifted instead closer to the CP when increasing the object deformability, probably to achieve more direct or stable control of the extremity that must be aligned with the hole.

For the long-hole insertion, the determinants for the selection of the GP were both the deformability and the accuracy of the task (Fig. 3b). The need to complete object insertion and minimize the traveling distance to the next GP prompts the participant to grasp the rigid object farther from the CP^29^. On the other side, the request for higher accuracy of the task limited the distance between the GP and the CP: indeed, if the GP–CP distance exceeds a certain threshold, the CP becomes more difficult to control. In fact, overall, while some participants chose to grasp the rigid object from the farthest extremity from the CP, the CP–GP distance never overcame the threshold of 205 mm for the intermediate object, and the threshold of 135 mm for the deformable one.

Moreover, the interaction of the accuracy and deformability was significant, in fact, the differences in the GP–CP distance between the tight hole and large hole were higher for the rigid and intermediate objects than the one for the deformable object. When grasping the rigid object, whatever the GP, the CP can still be controlled (though with less precision when GP goes farther from CP), leaving more variation margin for the choice of the GP. For the deformable object instead, the control of the CP is reasonably easy only grasping up to a certain distance from it (which limits the variation margin).

For the long-hole insertion, to maximize efficiency (the requirement was to complete the task accurately and as fast as possible), participants could choose between either grasping the object far from the CP and reducing the total number of re-grasps, or grasping the object near to the CP to align it with more accuracy and then re-grasping it multiple times. However, the farther the deformable object is grasped, the more difficult it is to achieve a precise alignment. On the other hand, the nearer the deformable object is grasped, the greater the number of re-grasps, and consequently the insertion time. Indeed, in the long-hole insertion, the insertion time increased with the deformability (Fig. 2f), as a consequence of the increasing number of re-grasps needed. Unlike the rigid object, for the deformable objects insertion time differed also for the required level of accuracy, which we explain with two hypotheses. First, the rigid object preserves part of the insertion movement impressed by the hand after the hand detaches, while the deformable tends to dump and store part of it as elastic energy. Second, there could have been slightly more friction for the deformable object than for the rigid one, because of both the Poisson effect (lateral expansion of the deformable object when it is compressed in the direction of the insertion) and the surface property of the deformable objects, which maybe was still a bit rougher than the rigid object (even though we reduced the difference by covering the objects with talcum powder before the experiment). Insertion time was indeed longer for the long tight hole. This longer time can be explained by the higher friction with the walls of the tight hole since those walls are closer to the object.

Concerning the strategy adopted most frequently by the participants for the long-hole insertion, for the rigid and intermediate objects almost all the participants required one re-grasp (Fig. 3c, Fig. S1a), even though the GP was not consistent among participants, as shown in Fig. S1b. For the deformable objects, there was less consistency among participants on the number of re-grasps used (Fig. S1a). Moreover, for the deformable object, participants selected the first GP in a narrower range than for the intermediate one (Fig. S1b). The strategy of the fastest five participants in manipulating the objects (sum of transport and insertion time) in a specific condition (Table SI, Supplementary material) was coherent with the one followed by the majority of the participants for the rigid and intermediate objects. For the deformable object, the fastest participants also chose as a strategy the maximization of the number of re-grasps (5 for the tight hole and 4 for the large). The possible reasons behind this choice are twofold: on one hand, the travel distance between each GP is minimized; on the other hand, the small distance between the subsequent re-grasping points reduces the likelihood of lateral inflections of the deformable object under compression (buckling effect).

### Accuracy and rigidity make alignment between the object and the hole more difficult

Various authors have studied the movement to place rigid objects into target areas with different tolerances^30–32^ and proved that transport time is longer when object-hole tolerance is smaller, that is for higher accuracy (and increasing difficulty) tasks. Other authors^33^ structured the experiment to demonstrate that the determinant for the increasing difficulty is the relationship between the size of the object and the target area width, rather than their absolute values. These results were confirmed by our findings for the insertion of the rigid object in the short hole. In addition, we proved that the same findings hold for the deformable objects: we found indeed that the transport time increases with increasing accuracy, across all levels of deformability.

First of all, we recall that, as shown in Fig. 3a,b, the deformable object was generally grasped nearer to the CP than the rigid object. This means that there might be small differences in the distance traveled by the hand during the transport phase. However, literature^34^ reported no significant differences for peg transport/alignment time when the traveled distance varied from 110 mm to 174 mm for movements of the right hand: even though the traveled distance in our experiment slightly changed due to the variation of the GP, since the amount of variation was smaller, we did not attribute the variation in transport time to these small changes in traveled distance, but to the different levels of deformability and accuracy required.

Present results showed that for the short-hole insertion, transport time decreases with increasing deformability and increases with accuracy (Fig. 2b). The possible explanations are two. Firstly, the rigid object requires compensatory movement to avoid, for example, the collision of the object with the table. For the deformable object, on the other hand, it is sufficient to move one end of the object without caring about the remaining part to complete the task. Secondly, the correct positioning of the object at the entrance of the hole is achieved more easily by exploiting the compliance of the deformable object.

For the long-hole insertion, transport time increased with accuracy and decreased with deformability as well (Fig. 2e). Moreover, moving from high to low accuracy, the transport time of rigid and intermediate objects registered a more consistent decrease than the deformable—the interaction between accuracy and deformability was significant. We hypothesized the following explanation, based on the evidence that when the GP–CP distance is shorter, a higher amount of hand rotation (requiring more time for execution) is needed to span the same path (Fig. 1d). This hand rotation was a combination of ulnar deviation and wrist supination movements. For the long hole, the GP–CP distance increased for lower accuracies. The amount of this increase is more consistent for the rigid and intermediate objects, and smaller (yet still significant) for the deformable object. A higher GP–CP distance might have produced faster CP movements. Hence, the increase in GP–CP distance might be responsible for the faster transport time of the rigid and intermediate objects in the low accuracy condition, which explains the higher difference in transport time between low and high accuracies for the rigid object than for the deformable, thus motivating the significance of the interaction. Although it is likely that the increase in GP–CP distance also complicates the final alignment of the CP, this might be less relevant when the tolerance is larger, as in this case. However, we stress this explanation has the nature of a hypothesis, whose proof can be obtained only by recovering the CP speed during the alignment phase of a low-accuracy insertion, when grasping the objects from different GPs.

### Effect of deformability and accuracy on the reaching movement

Literature exploring the reach-to-grasp phase has widely described how the kinematics of the reaching movement changes with several factors^6^, among which the size of the object to grasp^26,35–37^, the type of grasp adopted^35,37,38^, traveled distance, the direction of movement^26,37,39,40^ and task accuracy^41–44^. We investigated whether similar effects are detectable on the reaching velocity profiles when varying deformability.

#### Reaching duration

Literature^41–44^ provided evidence of the longer duration of the reaching movement for increasing accuracies of the end-goals. In our results, the reaching time was significantly longer for the insertion in the long tight hole than in the long large hole (Fig. 2d). However, the same results were not found for the short-hole insertion (Fig. 2a). Therefore, accuracy was accounted for only in the presence of a subsequent full insertion into the long hole, and not when the object had to be aligned and inserted into the short hole. According to the literature^41–44^, this indicates an influence of the end-goal on the reaching planning phase.

#### Time to peak velocity and amplitude of the peak

As concerns acceleration and deceleration time rates, we found an increasing acceleration time rate with increasing deformability (Fig. 4b,e), and consequently, the opposite for the deceleration time rate (Fig. 4c,f), which is complementary to it. Likewise, an increase in acceleration time rate was reported to happen in^35^ when increasing the size of the object (and switching from a precision to a power grasp).

We found no significant differences in peak velocity with increasing accuracy (Fig. 4a,d), confirming literature results^41,44^. We found no differences in deceleration time and deceleration time rate with increasing accuracy, in contrast with literature which reports their increase^44^. However, this literature compares precise tasks (placing or fitting) with non-precise ones (raising or throwing), while in our study we compared two insertions but with varying accuracy. Therefore the difference in accuracy between our tight-hole and large-hole insertion tasks might not be relevant enough to result in significant difference in terms of deceleration time.

Overall, in literature, the length of deceleration time is generally related to accuracy and precision requirements of the task, while the velocity peak has an inverse relationship with task accuracy^37^. Moreover, literature findings report increasing reaching time for more precise tasks^41–44^. Consequently, the higher velocity peak occurring proportionally later for the deformable object might suggest that reaching for the deformable object is perceived as a task requiring lower accuracy.

An alternative explanation could be that the more deformable the object, the less predictable its behavior during displacement. This could cause the agent to be unsure about the consequences of his/her actions, affecting the accuracy of his/her movement, as suggested by the literature^45^.

## Conclusions

Human behavior in manipulating deformable objects differs with the accuracy of the task and the deformability of the objects. Higher accuracy of the task or higher deformability of the object requires the grasp to be closer to the point that needs to be accurately controlled to complete the task. Interestingly, also the duration of the phases of the movement—as transport time or insertion time—are influenced by object deformability and accuracy of the task. Participants exploited the compliance of the deformable object to align it faster to the hole, but it required more re-grasps for the insertion, and consequently a longer insertion time. Moreover, deformability is accounted for since the planning phase of the transport, since differences were found also in the reaching kinematics.

This study paves the way for a series of studies in which the combination of the variation of attributes of the object deformability other than the bending deformability (i.e. compression properties), or other dependent variables (i.e. the different direction of movements, different end-goals, learning effect from the first to the last repetition) might be investigated. Enriching the discussion on human behavior when manipulating deformable objects increases the understanding of how humans manage the trade-off between time and accuracy according to specific requirements in terms of efficiency. Moreover, this knowledge enables the emulation of human manipulation behavior when programming robotic agents. The relevance of this emulation becomes even more important when robots should collaborate with humans: in fact, human’s sense of collaboration is enhanced if the agent acts with a more predictable or legible behavior.

For these reasons, we summarize here the principles for a human-inspired robotic planner for deformable object manipulation that stem from the findings of this study. These principles consider the tasks of grasping an object and assembling it.

- The grasping point on a rigid object should occur between the center of mass and the extremity of the object which is not involved in the assembly, to leave the assembly region (where the control point is) unobstructed. The higher the accuracy of the assembly, the closer the grasp should be to the control point for oblong objects: even if the robotic positioning is accurate, this choice reduces the effect of the moments generated by the contact with the environment on the grasp stability.
- The grasping point on a deformable object should be selected considering a compromise between leaving the assembly area unobstructed and staying close to the control point so that it is reasonably rigidly linked to the end effector.
- To minimize the completion time, the planning algorithm should minimize the number of re-grasps: in fact, the strategy that was most frequently chosen by human participants who are capable of seamless grasp replanning foresaw one single re-grasp for the rigid object and few for the deformable one. Also, replanning a grasp in robotic manipulation is generally more complex than achieving precise movements. This principle holds under the assumption that in-hand manipulations require complex planning in robotics, or may not even be possible with simpler types of grippers.
- As concerns the human-inspired motion, which is important in collaborative robotics (i.e. contributing to non-verbal communication^46,47^), the transport of the object toward the inlet of the assembly in case of a more precise task should be generally slower than for a non-precise task. Moreover, the movement of reaching for deformable objects should feature higher velocity peaks occurring proportionally later than for the rigid ones (as it happens for example when reaching for objects to perform non-precise tasks).

## Materials and methods

### Setup

The setup included three objects shaped as parallelepipeds with squared sections (300 mm × 22 mm × 22 mm) characterized by noticeably different levels of bending deformation. Two parallelepipeds were deformable and made of two different silicones: Smooth-On^®^ DragonSkin 10 for the object labeled as intermediate, and Smooth-On^®^ Ecoflex 0030 for the object labeled as deformable. The third parallelepiped was rigid and was made of polyoxymethylene. Since the weight plays a role in the selection of the grasping point, the rigid parallelepiped was hollowed out to achieve comparable weight with the silicone parallelepipeds (approximately 155 g). The rigid object was black, and the color intensity of the other two objects faded out with increasing deformability along a scale of greys. Talcum powder was put on the silicone parallelepipeds surface before each experiment, to reduce the difference in friction with the rigid one.

A schema of the setup is presented in Fig. 1a. The setup includes a table fully covered with millimeter paper: the participant sits centering their body to the middle line of the table. The hand area, namely the starting and ending position of the hand of the participant, lies on the right side of the table, 200 mm far from the middle line, and 100 mm far from the edge of the table where the participant sits. The object area, namely the area where the object is presented on the table, lies on the right side of the table, 200 mm far from the middle line and 250 mm far from the edge where the participant sits. At a distance of 400 mm from the edge of the table there was a black panel with a small window that allowed the participant to see a single hollow cavity, in which he was asked to insert the object. The window was placed on the right side of the table, 200 mm far from the middle line, at a height of 50 mm. This height allowed the participant to leave the object and re-grasp it again as many times as needed and avoided the table obstructing finger movements. As previously mentioned, the window in the black panel showed one hollow cavity at a time, but behind the black panel, there was a box with a total of four hollow cavities (dubbed “holes”). A robotic arm (which was hidden by the panel as well) moved the box to align one of the four holes with the window on the panel, to make it visible to the participant. Two holes were 10 mm deep, and had a squared section of side 23.1 mm (short tight hole) and 31.4 mm (short large hole) respectively; the other two holes were 240 mm deep and had a squared section of side 23.1 mm (long tight hole) and 31.4 mm (long large hole) respectively. The sizes of the holes were selected to achieve indexes of difficulty 8 bits (tight holes) and 5 bits (large holes) as defined by Fitt’s Law^48,49^.

An LED in correspondence to the window in the black panel notified the participant that the insertion had been completed.

The setup embeds sensors to detect task execution times. In particular, there were:

- a force sensing resistor (FSR^®^400, Interlink Electronics) in the hand area, to detect the moment in which the hand lifts from the starting position;
- a load cell (OnRobot, HEX-E sensor) supporting the object area to detect the moment in which the object is lifted;
- an infrared sensor (Omron, B5W-LB2101-1) at the inlet of each hole, to detect the moment in which the object enters the hole;
- a mechanical switch (Omron SS-01GL13-ET) at the bottom of each hole, to detect the completion of the insertion of the object.

The setup also includes a marker-based motion capture system (OptiTrack V120:Trio, NaturalPoint, Inc.) to record kinematic data: for this purpose, a support that mounted four reflective markers was anchored to the wrist of the participant. In addition, two video cameras recorded the participant’s hand from two different perspectives, and those videos served to measure the distances of the thumb and index fingertips (the grasping point) from the extremity of the object that was inserted first (the control point). A speaker emitted an acoustic sound as a “go” signal for the participant.

### Experimental protocol

#### Participants

A number of 19 right-handed participants (9 females, 28 ± 3 y.o.) were enrolled to participate in the experiment. The study followed the Declaration of Helsinki and informed consent was obtained from each participant before starting the experiment. This study was approved by the local ethical committee of the Scuola Superiore Sant’Anna, Pisa, Italy (approval n. 21/2022).

#### Experimental procedure

The experiment consisted of 2 steps. Step 1 (about 10 min) allowed the participant to become familiar with the objects, to understand how deformable they were (free exploration lasted about 1 min), and to practice the insertion task with the rigid object and the tight long hole, using a tri-digital grasp (thumb on one side of the object, index and middle fingers on the opposite side). If the participant happened to leave the object before the insertion was completed, she/he was instructed to re-grasp the object with a tri-digital grasp again.

Step 2 (about 60 min) consisted of the execution of the insertion tasks in 12 conditions. Each condition was characterized by a fixed deformability of the object, a fixed depth, and a tolerance of the hole. Conditions order was randomized among participants. When a condition was set up (one object with a certain deformability, a hole with certain tolerance and depth), the participant was asked to try the specific condition as many times as needed, to become familiar with it. We allowed this familiarization phase before recording the movements to capture the optimal strategy of the participant for each specific condition. After familiarization, in a single condition, each participant was asked to perform 12 insertions of the same object into the given hole. If the condition involved the intermediate or deformable object, before starting the 12 insertions, the participant performed 3 insertions with the rigid object in the given hole as washout repetitions. At the beginning of each insertion, the participant found one of the objects in the object area on the table. The hole to be used in the specific condition was the only one shown in the window in the black panel. The participant started with his hand on the hand area (thumb, index, and middle fingers in contact with the table). The participant was instructed to perform the following insertion sequence as fast as possible, using his right hand. To perform an insertion sequence, the participant had to:

1. Position her/his right hand in the hand area and wait for the “go” signal sound;
2. Pick the object with a tri-digital grasp;
3. Insert the object up to the bottom of the hole, until seeing the LED which indicates task completion lighting up;
4. Position her/his right hand back to the hand area. At the end of each insertion sequence, the experimenter re-placed the object in the object area on the table.

To encourage the execution as fast as possible, participants were told that any trial taking too long would have been considered failed and excluded. The participants were not allowed to use any in-hand manipulation during the whole insertion sequence: since some in-hand manipulations can be considered re-grasps, excluding those ensures a comparable number of pure re-grasps among participants. Moreover, we aimed to draw insights for robotic manipulation that apply also to grippers which are way less dexterous than the human hand.

The whole experiment lasted no longer than 75 min, including giving instructions and filling in the consent forms.

#### Data extraction

Execution times (reaching, transport, and insertion times) were retrieved by computing differences between the time instants identified by the sensors in the setup (see “Setup” paragraph). GP–CP distance was retrieved by looking at the videos of the video cameras, and manually taking note of the distances of thumb and index fingertips from the face of the parallelepiped which was then inserted first in the hole, that is the CP. The millimeter paper which fully covered the table was taken as a reference for measures (resolution: 5 mm). Then, the mean value between the distances of the thumb and the index fingertips from the CP was calculated and considered as GP–CP distance. The number of re-grasps was retrieved by looking at the video of the whole scene and manually taking notes.

As concerns parameters extracted from kinematics data, we considered only 17 participants, due to some problems with the tracking device. Before computing parameters from the wrist velocity profile during reaching, we also had to exclude other 6 repetitions of the insertion in total, because the velocity profile of those repetitions did not show the standard bell-shaped profile of a typical reaching movement: a non-bell-shaped velocity profile precludes the computation of the chosen parameters. We computed the velocity profile by differentiating the recorded position of the wrist. Acceleration time was computed from the smoothed wrist velocity profile during reaching as the time between the start of the movement and the time at which peak velocity was reached (Fig. 1c). Deceleration time was computed from the smoothed wrist velocity profile as the time between the peak velocity and the next velocity minimum (Fig. 1c). We also computed the reaching phase duration from the velocity profile as the sum of acceleration and deceleration time, and we obtained the acceleration (or deceleration) time rates by dividing acceleration (or deceleration) time per the reaching phase duration.

All the values, namely execution times (reaching, transport, insertion), GP–CP distance, and reaching velocity parameters (peak velocity, acceleration and deceleration time and time rates, and reaching phase duration) were computed for each repetition and condition using MATLAB^®^. For each participant and condition, we removed outlier values (outside the interval *mean* ± *2 · standard deviation*) among the 12 repetitions of the insertion, and we computed the mean value of the remaining repetitions.

#### Most frequently adopted and fastest strategy for the long-hole insertion

We identified the number of re-grasps most frequently adopted by the participants when performing the long-hole insertion by plotting the number of participants that had selected a specific number of re-grasps for each of the 6 conditions (long tight and large holes with rigid, intermediate, and deformable object). We extracted then the subset of GP–CP distances of participants who executed the task with the number of re-grasps most frequently adopted in the specific condition, and computed mean and standard deviation (the number of samples was 15, 11, and 9 for the rigid, intermediate and deformable object in the tight-hole insertion, 16, 12, and 10 for the rigid, intermediate and deformable object in the large-hole insertion). We identified the participants who were the fastest on average in transporting and inserting the object for the long-hole insertion by computing the sum of the mean values of transport and insertion time, retrieving the 5 participants with the lower values for manipulation (transport plus insertion) time in each of the 6 conditions, and reporting the number of re-grasps and mean GP-CP distance adopted.

### Statistical analyses

We performed statistical analyses separately for the short hole (6 conditions) and long hole (6 conditions), using IBM^®^ SPSS^®^ software. We tested the effect of deformability, accuracy, and of their interaction on the data extracted. When the hypothesis that “the data for each condition were normally distributed” was refused for none of the six conditions (Kolmogorov–Smirnov test with Lilliefors correction, α = 0.01), two-way repeated measures ANOVA two-tailed test with α = 0.05 was performed. Post-hoc tests with Bonferroni correction were performed whenever one of the factors or their interaction was significant. This was the case for reaching and transport time, insertion time in the long hole, and all the parameters extracted from the wrist velocity profile. When the hypothesis that “the data for each condition were normally distributed” was refused for any of the six conditions (Kolmogorov–Smirnov test with Lilliefors correction, α = 0.01), we performed a two-way non-parametric test following the method in^50,51^, which consists briefly in using a non-parametric test on aggregated data to test for significance of each of the two factors and their interaction. As non-parametric tests, we choose the Friedman test with α = 0.05 when testing differences between three paired groups, the T-Wilcoxon Signed Ranks test with α = 0.05 when testing differences between two paired groups whose distribution was symmetric (|γ| < 1, where γ is the skewness index), or Sign test with α = 0.05 when testing differences between two groups whose distribution was not symmetric. Whenever one of the factors or their interaction was significant, we performed post-hoc non-parametric tests with Bonferroni correction. This was the case for the insertion time in the short hole, for the GP–CP distance, and for the number of re-grasps.

Finally, we compared the dispersion of the GP–CP distance for intermediate and deformable objects for the participants who executed the task with the most frequently adopted number of re-grasps by using Kruskal–Wallis test with α = 0.05. In this case, the number of samples was 15, 11, and 9 for rigid, intermediate, and deformable objects respectively in the tight-hole insertion, and 16, 12, and 10 for rigid, intermediate, and deformable objects respectively in the large-hole insertion.

### Supplementary Information

Supplementary Information.

Supplementary Video 1.

## Data Availability

The datasets generated and analyzed during the current study are available in the “sssa_deformability” repository https://github.com/sssa-human-robot-interaction-lab/sssa_deformability.git.

## References

[1] Danion F, Diamond JS, Flanagan JR (2012). The role of haptic feedback when manipulating nonrigid objects. J. Neurophysiol..

[2] Yin H, Varava A, Kragic D (2021). Modeling, learning, perception, and control methods for deformable object manipulation. Sci. Robot..

[3] Billard A, Kragic D (2019). Trends and challenges in robot manipulation. Science.

[4] Makris S, Kampourakis E, Andronas D (2022). On deformable object handling: Model-based motion planning for human-robot co-manipulation. CIRP Ann..

[5] Kruse, D.; Radke, R.J.; Wen, J.T. Collaborative Human-Robot Manipulation of Highly Deformable Materials. In *Proc IEEE Int Conf Robot Autom* **2015**, 3782–3787. 10.1109/ICRA.2015.7139725 (2015).

[6] Castiello U, Dadda M (2019). A review and consideration on the kinematics of reach-to-grasp movements in macaque monkeys. J. Neurophysiol..

[7] Feix T, Bullock IM, Dollar AM (2014). Analysis of human grasping behavior: Object characteristics and grasp type. IEEE Trans. Haptics.

[8] Feix T, Bullock IM, Dollar AM (2014). Analysis of human grasping behavior: Correlating tasks, objects and grasps. IEEE Trans. Haptics.

[9] Cini F, Ortenzi V, Corke P, Controzzi M (2019). On the choice of grasp type and location when handing over an object. Sci. Robot..

[10] Huang Y, Bianchi M, Liarokapis M, Sun Y (2016). Recent data sets on object manipulation: A survey. Big Data.

[11] Balaguer B, Carpin S (2011). Combining imitation and reinforcement learning to fold deformable planar objects. IROS.

[12] Li Z, Miao F, Yang Z, Chai P, Yang S (2019). Factors affecting human hand grasp type in tomato fruit-picking. Comput. Electron. Agric..

[13] Verleysen A, Biondina M, Wyffels F (2020). Video dataset of human demonstrations of folding clothing for robotic folding. Int. J. Rob. Res..

[14] Bullock IM, Feix T, Dollar AM (2013). Finding small, versatile sets of human grasps to span common objects. Proc. IEEE Int. Conf. Robot. Autom..

[15] Feix T, Romero J, Schmiedmayer HB, Dollar AM, Kragic D (2016). The GRASP taxonomy of human grasp types. IEEE Trans. Hum. Mach. Syst..

[16] Nayeem R, Bazzi S, Sadeghi M, Hogan N, Sternad D (2021). Preparing to move: Setting initial conditions to simplify interactions with complex objects. PLoS Comput. Biol..

[17] Dingwell JB, Mah CD, Mussa-Ivaldi FA (2004). Experimentally confirmed mathematical model for human control of a non-rigid object. J. Neurophysiol..

[18] Svinin M, Goncharenko I, Kryssanov V, Magid E (2019). Motion planning strategies in human control of non-rigid objects with internal degrees of freedom. Hum. Mov. Sci..

[19] Heald JB, Ingram JN, Flanagan JR, Wolpert DM (2018). Multiple motor memories are learned to control different points on a tool. Nat. Hum. Behav..

[20] Landelle C, Montagnini A, Madelain L, Danion F (2016). Eye tracking a self-moved target with complex hand-target dynamics. J. Neurophysiol..

[21] Dingwell JB, Mah CD, Mussa-Ivaldi FA (2002). Manipulating objects with internal degrees of freedom: Evidence for model-based control. J. Neurophysiol..

[22] Ortenzi V, Controzzi M, Cini F, Leitner J, Bianchi M, Roa MA, Corke P (2019). Robotic manipulation and the role of the task in the metric of success. Nat. Mach. Intell..

[23] Ortenzi V, Cini F, Pardi T, Marturi N, Stolkin R, Corke P, Controzzi M (2020). The grasp strategy of a robot passer influences performance and quality of the robot-human object handover. Front. Robot. AI.

[24] Lederman SJ, Wing AM (2003). Perceptual judgement, grasp point selection and object symmetry. Exp. Brain Res..

[25] Endo S, Wing AM, Bracewell RM (2011). Haptic and visual influences on grasp point selection. J. Mot. Behav..

[26] Paulignan Y, Frak VG, Toni I, Jeannerod M (1997). Influence of object position and size on human prehension movements. Exp. Brain Res..

[27] Paulun VC, Kleinholdermann U, Gegenfurtner KR, Smeets JBJ, Brenner E (2014). Center or side: Biases in selecting grasp points on small bars. Exp. Brain Res..

[28] Klein LK, Maiello G, Paulun VC, Fleming RW (2020). Predicting precision grip grasp locations on three-dimensional objects. PLoS Comput. Biol..

[29] Jovanovic B, Schwarzer G (2011). Learning to grasp efficiently: The development of motor planning and the role of observational learning. Vis. Res..

[30] Annett J, Golby CW, Kay H (1958). The measurement of elements in an assembly task—The information output of the human motor system. Q. J. Exp. Psychol..

[31] Annett J, Annett M, Hudson PTW, Turner A (1979). The control of movement in the preferred and non-preferred hands. Q. J. Exp. Psychol..

[32] Milner TE, Ijaz MM (1990). The effect of accuracy constraints on three-dimensional movement kinematics. Neuroscience.

[33] Srinivasan D, Martin B (2008). Object and target size interactions in placement tasks. Proc. Hum. Fact. Ergon. Soc. Annu. Meet..

[34] Tochio, K., Kimura, D., Kinoshita, H., Ryuhei, O. & Fukui, T. Independent Evaluation of peg travel and reach movement time using a newly developed nine-hole pegboard. 10.21203/RS.3.RS-1270835/V1 (2022).

[35] Gentilucci M, Castiello U, Corradini ML, Scarpa M, Umiltà C, Rizzolatti G (1991). Influence of different types of grasping on the transport component of prehension movements. Neuropsychologia.

[36] Jakobson LS, Goodale MA (1991). Factors affecting higher-order movement planning: A kinematic analysis of human prehension. Exp. Brain Res..

[37] Castiello U (1996). Grasping a fruit: Selection for action. J. Exp. Psychol. Hum. Percept. Perform..

[38] Castiello U, Bennett KMB, Stelmach GE (1993). The bilateral reach to grasp movement. Behav. Brain Res..

[39] Paulignan Y, MacKenzie C, Marteniuk R, Jeannerod M (1991). Selective perturbation of visual input during prehension movements. 1. The effects of changing object position. Exp. Brain Res..

[40] Connolly JD, Goodale MA (1999). The role of visual feedback of hand position in the control of manual prehension. Exp. Brain Res..

[41] Armbrüster C, Spijkers W (2006). Movement planning in prehension: Do intended actions influence the initial reach and grasp movement?. Motor Control.

[42] Ansuini C, Santello M, Massaccesi S, Castiello U (2006). Effects of end-goal on hand shaping. J. Neurophysiol..

[43] Ansuini C, Giosa L, Turella L, Altoè G, Castiello U (2008). An object for an action, the same object for other actions: Effects on hand shaping. Exp. Brain Res..

[44] Marteniuk RG, MacKenzie CL, Jeannerod M, Athenes S, Dugas C (1987). Constraints on human arm movement trajectories. Can. J. Psychol..

[45] Gamberini L, Carlesso C, Seraglia B, Craighero L (2013). A behavioural experiment in virtual reality to verify the role of action function in space coding. Vis. Cogn..

[46] Sciutti A, Patanè L, Nori F, Sandini G (2014). Understanding object weight from human and humanoid lifting actions. IEEE Trans. Auton. Ment. Dev..

[47] Lastrico, L., Duarte, N. F., Carfí, A., Rea, F., Mastrogiovanni, F., Sciutti, A. & Santos-Victor, J. If You Are Careful, So Am I! How Robot Communicative Motions Can Influence Human Approach in a Joint Task. *Lecture Notes in Computer Science (including subseries Lecture Notes in Artificial Intelligence and Lecture Notes in Bioinformatics)**13817 LNAI*, 267–279. 10.1007/978-3-031-24667-8_24 (2022).

[48] Massimino MJ, Sheridan TB (2016). Teleoperator performance with varying force and visual feedback. Hum. Factors.

[49] Fitts PM (1954). The information capacity of the human motor system in controlling the amplitude of movement. J. Exp. Psychol..

[50] Koch GG (1970). The use of non-parametric methods in the statistical analysis of a complex split plot experiment. Biometrics.

[51] Landenna, G. & Marasini, D. Metodi Statistici Non Parametrici. *La Nuova scienza. Serie di scienze sociali* (1990).

