# Supplementary material for: Human manipulation strategy when changing object deformability and task properties
Source: Sci Rep. 2024 Jul 9;14:15819. doi: 10.1038/s41598-024-65551-x (PMC11233673; doi:10.1038/s41598-024-65551-x)
Supplement: Supplementary file 1 — Supplementary Information. [file 41598_2024_65551_MOESM1_ESM.docx]

Supplementary Materials

Fig. S1 Grasping behavior adopted by the participants.

Fig. S2 Parameters from wrist reaching velocity profile: reaching time (s), and acceleration and deceleration time (s), for insertions in the short and long holes.

Table SI Strategy adopted by the participants who were the fastest in transporting and inserting the object.

Table SII Statistical results for the reaching time (s) extracted from the wrist velocity profile, and acceleration and deceleration times (s).

Movie S1 Summary of the experimental protocol and main results, with examples of the execution of the insertion task.

**Figures:**


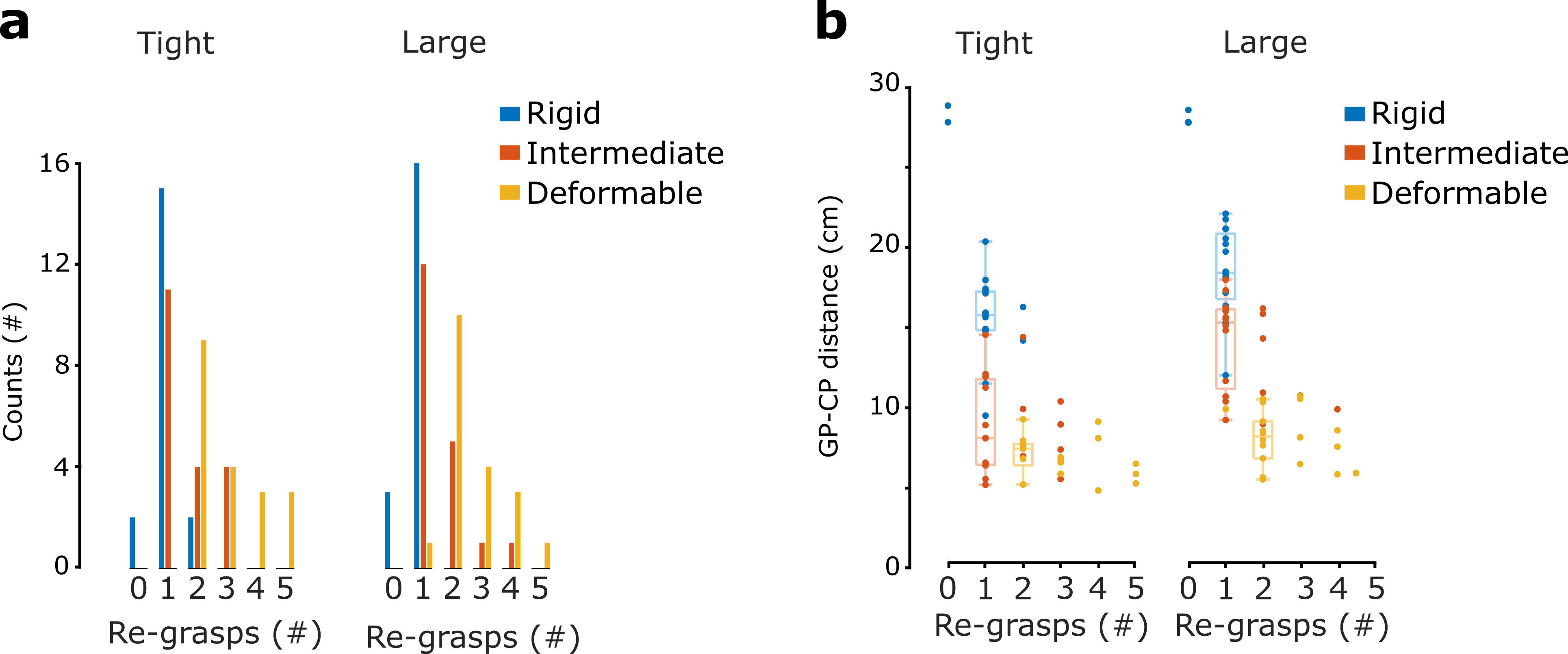


**Fig. S1. Grasping behavior adopted by the participants.** (**a**) The histograms show how many participants used a given number of re-grasps for each condition. (**b**) Each dot represents one participant inserting one of the objects (see color legend) in the long tight or large hole. The dot indicates the median number of re-grasps (horizontal axis) and mean grasping point – control point (GP-CP) distance for the first grasp (vertical axis) chosen by the participant for a given object deformability. The boxplots describe the distribution of GP-CP distance for the most frequently adopted combination of GP-CP distance and number of re-grasps for each condition.


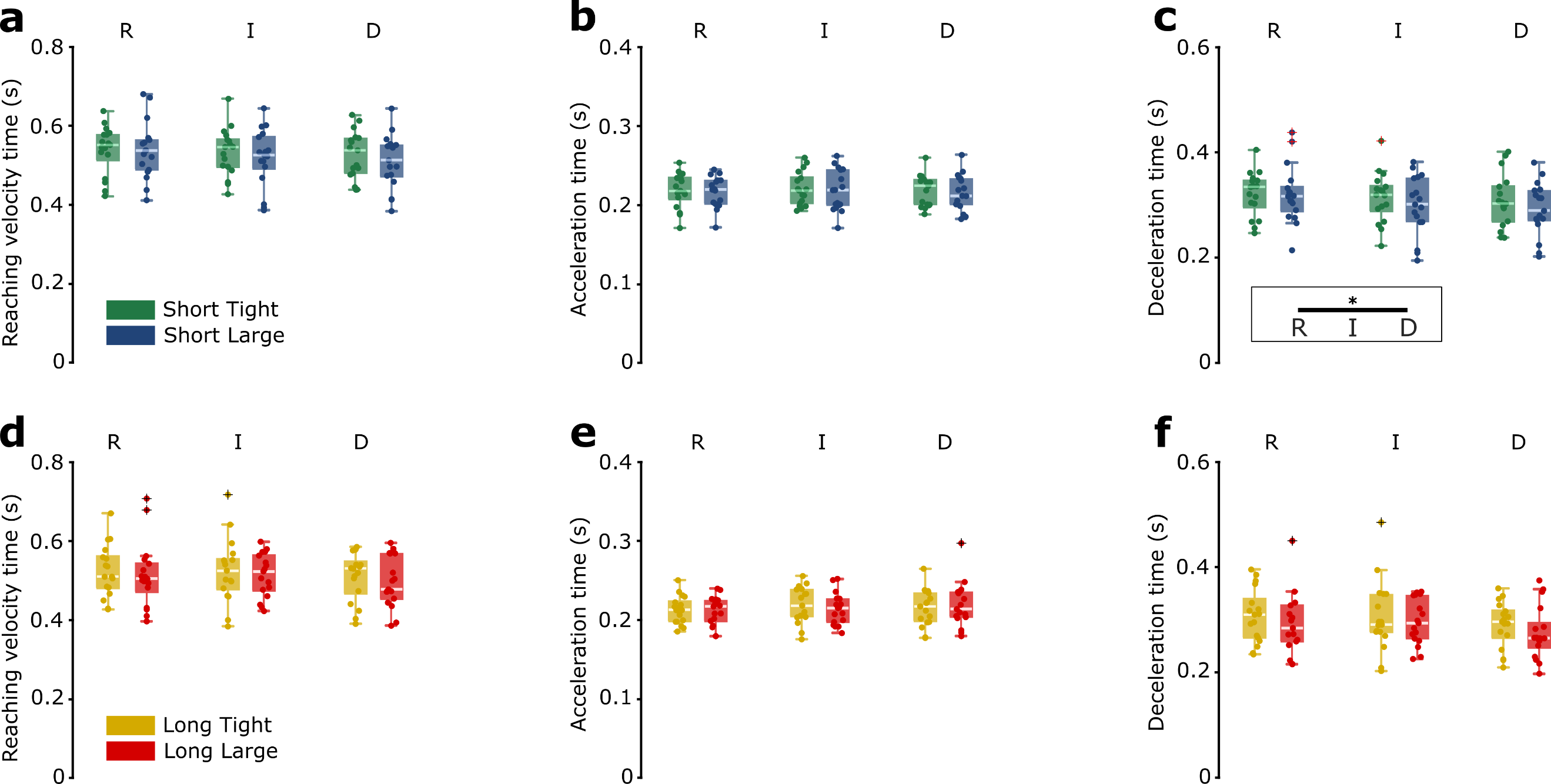
**Fig. S2. Parameters from wrist reaching velocity profile: reaching time (s), and acceleration and deceleration time (s), for insertions in the short and long holes.** Boxplots show the distribution (median, IQR, max/min value, outliers) of the mean reaching kinematic parameters, for the short- and long-hole insertion for each participant across the conditions of hole tolerance (T tight, L large) and object deformability (R rigid, I intermediate, D deformable). The dots represent the mean reaching time, and acceleration and deceleration time, for the short- and long-hole insertion for each participant in a specific condition. Horizontal bars represent significant differences obtained with post-hoc tests, in black between deformability or accuracy levels (within the boxes), in gray between experimental conditions; asterisks refer to the Bonferroni adjusted *p*-values within the following ranges: * for *p_adj_* < 0.05, ** for *p_adj_* < 0.01, *** for *p_adj_* < 0.001. (**a**) Mean reaching time (extracted from wrist velocity profile), (**b**) Mean acceleration time, and (**c**) Mean deceleration time for the short-hole insertion. (**d**) Mean reaching time (extracted from wrist velocity profile), (**e**) Mean acceleration time, and (**f**) Mean deceleration time for the long-hole insertion.

**Tables:**

**Table SI. Strategy adopted by the participants who were the fastest in transporting and inserting the object.** Cells are highlighted in light blue when either (1) the number of re-grasps corresponded to the most frequently chosen one in the same condition, or (2) when the GP-CP distance falls within the range mean ± standard deviation identified for GP-CP distance when analyzing the most frequently adopted strategy (more details in the paragraph *Most frequently adopted and fastest strategy for the long-hole insertion*). Cells are highlighted in blue when both conditions (1) and (2) are verified.

|  | **Tight hole** | | | |  |  | **Large hole** | | | |
| --- | --- | --- | --- | --- | --- | --- | --- | --- | --- | --- |
|  | Participant  ID # | # re-grasps | GP-CP dist. (cm) | Transp. + Insert. time (s) |  |  | Participant  ID # | # re-grasps | GP-CP dist. (cm) | Transp. + Insert. time (s) |
| **Rigid** | 3 | 1 | 16 | 1.580 |  | **Rigid** | 7 | 1 | 18 | 1.406 |
|  | 4 | 0 | 29 | 1.594 |  |  | 3 | 1 | 21 | 1.521 |
|  | 19 | 2 | 16 | 1.726 |  |  | 10 | 1 | 15 | 1.607 |
|  | 6 | 1 | 15 | 1.986 |  |  | 15 | 1 | 20 | 1.771 |
|  | 5 | 1 | 10 | 2.538 |  |  | 5 | 1 | 12 | 1.874 |
| **Intermediate** | 15 | 1 | 7 | 1.438 |  | **Intermediate** | 16 | 1 | 12 | 1.665 |
|  | 7 | 1 | 9 | 1.584 |  |  | 12 | 2 | 14 | 1.810 |
|  | 19 | 2 | 7 | 2.145 |  |  | 7 | 1 | 16 | 1.816 |
|  | 17 | 1 | 12 | 2.264 |  |  | 9 | 1 | 15 | 1.997 |
|  | 14 | 1 | 15 | 2.811 |  |  | 14 | 1 | 17 | 2.003 |
| **Deformable** | 8 | 5 | 7 | 2.098 |  | **Deformable** | 8 | 4 | 6 | 1.460 |
|  | 7 | 2 | 8 | 2.165 |  |  | 4 | 4.5 | 6 | 1.838 |
|  | 4 | 5 | 5 | 2.197 |  |  | 18 | 2 | 8 | 1.931 |
|  | 18 | 2 | 7 | 2.436 |  |  | 10 | 2 | 6 | 2.065 |
|  | 9 | 2 | 8 | 2.553 |  |  | 6 | 2 | 6 | 2.363 |

**Table SII.** **Statistical results for the reaching time (s) extracted from the wrist velocity profile, and acceleration and deceleration times (s)**. Results of ANOVA tests (F) and its post-hoc comparisons. For the post-hoc test, *p*-values (*p_adj_*) are adjusted with the Bonferroni correction. Significant results (*p* < 0.05) are highlighted in boldface. R: Rigid object, I: Intermediate object, D: Deformable object.

|  |  | | Reaching time | | Acceleration time | | Deceleration time | |
| --- | --- | --- | --- | --- | --- | --- | --- | --- |
|  |  | | Short hole | Long hole | Short hole | Long hole | Short hole | Long hole |
|  |  | | N = 17 | N = 17 | N = 17 | N = 17 | N = 17 | N = 17 |
| Main Effects | Deformability | | **F = 3.536**  ***p* = 0.041** | F = 1.130  *p* = 0.336 | F = 0.405  *p* = 0.670 | F = 1.377  *p* = 0.267 | **F = 4.756**  ***p* = 0.016** | F = 2.106  *p* = 0.138 |
|  | Accuracy | | F = 1.228  *p* = 0.284 | F = 2.880  *p* = 0.109 | F = 0.797  *p* = 0.385 | F = 0.115  *p* = 0.739 | F = 1.853  *p* = 0.192 | F = 2.618  *p* = 0.125 |
|  | Deformability  *Accuracy | | F = 0.440  *p* = 0.648 | F = 0.045  *p* = 0.956 | F = 0.003  *p* = 0.997 | F = 1.334  *p* = 0.278 | F = 0.701  *p* = 0.504 | F = 0.144  *p* = 0.866 |
| Post-hoc tests | Def. | R – I | *p_adj_* = 0.526 | - | - | - | *p_adj_* = 0.260 | - |
|  |  | R – D | *p_adj_* = 0.089 | **-** | **-** | **-** | ***p_adj_* = 0.036** | **-** |
|  |  | I – D | *p_adj_* = 0.475 | - | - | - | *p_adj_* = 0.685 | - |
